# Using self-collection HPV testing to increase engagement in cervical cancer screening programs in rural Guatemala: a longitudinal analysis

**DOI:** 10.1186/s12889-020-09478-8

**Published:** 2020-09-15

**Authors:** Anna Gottschlich, Alvaro Rivera-Andrade, Kristin Bevilacqua, Audrey R. Murchland, Ergest Isak, Christian S. Alvarez, Gina Ogilvie, Thomas E. Carey, Mark Prince, Michael Dean, Carlos Mendoza-Montano, Rafael Meza

**Affiliations:** 1 Department of Epidemiology, School of Public Health, University of Michigan, Ann Arbor, MI USA; 2 Vancouver, Canada; 3 Institute of Nutrition of Central America and Panama-INCAP, Guatemala City, Guatemala; 4 Division of Cancer Epidemiology and Genetics, National Cancer Institute, Rockville, MD USA; 5 Clinical Prevention Services, BC Centre for Disease Control, Vancouver, BC Canada; 6 Department of Pharmacology, University of Michigan, Ann Arbor, MI USA; 7 Department of Otolaryngology, University of Michigan, Ann Arbor, MI USA; 8 Laboratory of Translational Genomics, Division of Cancer, Epidemiology and Genetics, National Cancer Institute, Gaithersburg, MD USA

**Keywords:** Cervical cancer, Human papillomavirus, Self-collection, Indigenous populations

## Abstract

**Background:**

Cervical cancer is a leading cause of death in low- and middle-income countries. Self-collection testing for human papillomavirus (HPV) is an alternative form of cervical cancer screening that can be completed privately and at home. Understanding how the use of HPV testing influences follow-up care in low-resourced settings is crucial before broad implementation. This study aimed to identify if access to self-collection HPV testing impacts participation in established cervical cancer screening programs among women in two rural communities in Guatemala.

**Methods:**

A cohort of 956 women was recruited in 2016 and followed for 2 years for the HPV Multiethnic Study (HPV MES). At baseline, women answered a questionnaire assessing cervical cancer screening history and were offered self-collection HPV testing. Women were re-contacted yearly to determine receipt of additional screening. Statistical changes in screening behavior before and throughout study participation, stratified by self-collection status, were assessed using McNemar pair tests for proportions. Alluvial plots were constructed to depict changes in individual screening behavior. The odds of changes in Pap-compliance (screened in past 3 years), given collection status, were assessed using multivariate logistic regressions.

**Results:**

Reported screening rates increased 2 years after enrollment compared to rates reported for the 3 years before study entry among women who collected a sample (19.1% increase, *p* < 0.05), received results of their test (22.1% increase, *p* < 0.05), and received positive (24.2% increase, *p* < 0.1) or negative results (21.7% increase, *p* < 0.05). However, most increases came from one community, with minimal changes in the other. The adjusted odds of becoming Pap compliant were higher for women who collected a sample vs. did not (OR: 1.48, 95% CI: 0.64, 3.40), received their result vs. did not (OR: 1.29, 95% CI: 0.52, 3.02), and received a positive result vs. negative (OR: 2.43, 95% CI: 0.63, 16.10).

**Conclusions:**

Participation in self-collection HPV testing campaigns may increase likelihood of involvement in screening programs. However, results varied between communities, and reporting of screening histories was inconsistent. Future work should identify what community-specific factors promote success in HPV testing programs and focus on improving education on existing cervical cancer interventions.

## Background

Cervical cancer in low- and middle-income countries (LMICs) accounted for approximately 90% of the 311,000 cervical cancer deaths worldwide in 2018 [1]. In contrast, since the introduction of cervical cancer screening, incidence and mortality rates have been relatively low in high-income countries (HICs) [2], demonstrating the potential to reduce the global burden of cervical cancer with improved screening. While screening has been implemented in LMICs, it has not reached the same success as in HICs [3]. Thus, there is a need for alternative prevention strategies in LMICs.

Guatemala is a middle-income country in Central America with a population of approximately 15 million [4]. Today, over 35% of the country’s total population is 25–54 years old (~ 2.6 million women), when women are at highest risk for cervical cancer development [4, 5]. Lack of screening among women in Guatemala [6] contributes to an exceptionally high cervical cancer incidence rate of 21.2 per 100,000 women, compared to 6.5 per 100,000 women in the United States, where screening is more common [7].

A number of barriers to screening have been identified in Guatemala. Existing screening options involve either cytological testing with a Papanicolaou (Pap) smear to identify abnormal cervical cells or visual inspection with acetic acid (VIA) to detect changes in keratinization on the cervix [8]. Pap smears often involve multiple doctor visits before receiving results, and due to the low sensitivity of the test, require frequent screening intervals [9]. VIA, while designed to be a “see-and-treat” procedure involving only one appointment, often still necessitates multiple visits and has both low sensitivity and poor inter-observer reliability [10–12]. For certain women, these screening methods are likely prohibitive, as language barriers and resource constraints are associated with decreased access to health care and specifically cervical cancer screening, both worldwide and particularly in Guatemala [13]. Recent analyses of nationally representative health survey data found that women who reported experiencing a barrier to health care (e.g. language, cost, and distance) were less likely to report prior cervical cancer screening [13].

High-risk human papillomavirus (HPV) is the cause of virtually all cervical cancers; though, the majority of infections do not lead to cervical cancer [1]. HPV testing has become an alternative primary cervical cancer screening option. The test for HPV is extremely sensitive, has high negative predictive value, and can be self-collected by a woman in her own home [14, 15]. If a woman tests positive for HPV, it is recommended she seek follow-up care from a medical professional; if she tests negative, it is recommended she retest in 3–5 years [16] or even longer [17]. As such, in resource-constrained settings, it is likely that even 1–2 lifetime HPV tests would significantly decrease rates of cervical cancer [18]. Self-collection HPV testing has been shown to be highly acceptable across many areas and cultures, including indigenous populations in Guatemala [19, 20]. Additionally, a pilot implementation trial for HPV self-collection testing concentrated in urban areas is being conducted by the Ministry of Health [21]. Though the scalability and long-term impacts of this type of program are still under investigation in low-resourced settings, the research that has been conducted shows promising results [22].

While barriers to screening may be overcome through HPV self-testing, considering the low rates of follow-up and treatment of positive Pap smears across Latin America [23–25], it is unclear if a primary HPV testing program in Guatemala would perform differently than conventional screening, particularly among those women who require follow-up care. Thus, rather than assessing the impact of primary HPV self-collection testing as a replacement for existing programs, we evaluated the effect of an HPV self-collection program on future engagement with the existing national screening program. Based on prior research [26], we hypothesized that women who have been introduced to cervical cancer screening through HPV self-collection programs would be more likely to have continued engagement with cervical cancer screening due to increased knowledge of HPV and cervical cancer prevention. This may empower women to advocate to receive regular preventative screening. Further, the potential for primary screening to be performed in the privacy of one’s own home may improve future screening behavior by reducing negative feelings associated with cervix screening [19, 20]. We also hypothesized that the novelty of a new technology coupled with the ability of this technology to test for a virus that is a known cause of cancer may make HPV testing more appealing than conventional methods (i.e. testing positive for HPV may prompt women to seek further screening).

We used data from the HPV multi-ethnic study (HPV MES) to compare the prevalence of reported cervical cancer screening before and after access to study-provided HPV self-collection testing to assess whether HPV self-collection testing increased participation in available screening programs in the community as a proxy for the overall success of the intervention.

## Methods

HPV MES began in 2015 with a pilot study (*N* = 202) offering HPV self-collection testing to evaluate if this would be an acceptable and impactful method of primary cervical cancer screening in multi-ethnic, rural areas of Guatemala. The pilot cross-sectional study demonstrated high acceptability of self-collection [19, 20], so we proceeded to recruit a cohort in 2016 to investigate the impact of a HPV self-collection program on rates of future screening and follow-up. Women in the cohort study were offered HPV self-collection testing at baseline and then were re-contacted at two annual interviews to identify continued interactions with existing screening programs, creating three waves of data collection: baseline (survey and optional HPV testing), follow-up in 2017 (FU1: survey), and follow-up in 2018 (FU2: survey). Surveys were specifically developed for this study and were based on the pilot survey which was created using the STEPwise Approach to Surveillance (STEPS) survey [27] and the University of Michigan’s Michigan HPV and Oropharyngeal Cancer study [28]. Blank copies of the baseline, FU1, and FU2 questionnaires can be found in the Supplementary Material (Survey A1, A2, A3).

HPV MES has been previously described in detail along with baseline acceptability results [19, 20]. Briefly, the cohort included 956 women from two distinct communities in rural Guatemala: Santiago Atitlan, Sololá (*N* = 500) and Livingston, Izabal (*N* = 456). In Santiago Atitlan, over 95% of participants identified as Tz’utujil Mayan, while in Livingston, 25% identified as Ladino (of Spanish or mixed descent), 32% as Garífuna (of Afro-Caribbean descent), and 42% as Q’eqchi Mayan [19]. For these analyses, the data was subset to participants between the ages of 25 and 54 (*N* = 438 in Santiago, *N* = 322 in Livingston), which is the age group eligible for cervical cancer screening according to guidelines in Guatemala.

### Data

At baseline, women completed an in-home comprehensive questionnaire facilitated by community health workers (CHWs) and were offered a self-collection HPV test. After completion of the questionnaire, regardless of decision to self-collect, all participants received a voucher for free screening in their local private clinic. This was in addition to the free screening available in their local public clinics. Notably, while nearly all age-eligible Santiago participants chose to self-collect (*N* = 410, 94%), only 169 (53%) of age-eligible Livingston participants self-collected [19]. However, among those who collected, over 80% in both communities found the test comfortable and easy to use, and 95% were willing to continue using HPV testing as cervical cancer screening, demonstrating a high acceptability of the intervention [19]. Test results were returned to 347 (85%) and 113 (67%) of participants in Santiago and Livingston, respectively, within 3 months of recruitment, along with recommendations for next steps. Positive results were returned in-person by a physician and negative results were returned over the phone by a CHW. Of the participants who were not immediately contacted, 40 and 34 in Santiago and Livingston, respectively, had inconclusive test results that were retested throughout follow-up, and the remaining 23 and 22, respectively, were unable to be reached. At study conclusion in 2018, participants with initially inconclusive results were contacted and attempts were made to contact those who were previously unreachable.

At baseline, women were consented to participate in the baseline survey and HPV testing and separately consented to follow-up contact. Women who consented to follow-up were re-contacted by phone at FU1 and FU2. During follow-up waves, all consenting participants (regardless of decision to self-collect) were asked to complete a short questionnaire about any cervical cancer screening or follow-up care that they received that follow-up year.

### Variable creation and statistical analysis

Any reported screen over follow-up was considered an initial screen (i.e. not a follow-up test in accordance with known prior screening results). At the time of the study, HPV testing was not approved for clinical use in Guatemala, so all women, regardless of HPV status, were referred for screening to the national screening program. It is possible that women who were HPV positive provided these results to their health care worker during screening, but, as the data is self-reported, we have no way to verify this. In practice, initial screening and follow-up testing would appear identical, with both involving either cytology or VIA.

We measured the prevalence of ever-screened for cervical cancer, which is an established measure of performance of screening programs [29], as well as the proportion of women compliant with country-specific screening guidelines [8, 30]. Screening compliance was defined as screening in the past 3 years, in accordance with the Guatemalan Ministry of Health recommendation. All data was self-reported [29], and we were unable to confirm receipt of screening after study participation through health clinics. Ever screened and screening compliance were calculated at each study wave for age (25–39/40–54), location (Santiago/Livingston), ethnicity (Tz’utujil or Ladino in Santiago; Q’echchi, Ladino, or Garifuna in Livingston), literacy (yes/no), education level (less than primary, primary or secondary, more than secondary), and the following, non-exclusive, six groupings: 1) those who completed self-collection, 2) those who did not complete self-collected, 3) those who received the results of their HPV test, 4) those who did not received results, and, of those who had received results, 5) those who tested positive and 6) those who tested negative.

We then investigated changes in screening behavior between that reported at baseline (for the 3 years prior to study participation) and that reported at FU1 (for the year post-entry) and FU2 (for the 2 years post-entry). We stratified the population at baseline by the above-described six HPV test collection-related groupings and location (Santiago/Livingston) and calculated the percent who reported screening prior to and post-baseline entry into the study. We used McNemar tests for paired proportions to assess statistical changes between the percent of women who had screened in the 3 years before baseline and the percent who screened during the first year and first 2 years after study entry for each group. This analysis was repeated after stratifying by participants who self-identified as literate (“I can read and write.”) versus those who identified as illiterate (“I cannot read and/or write.”).

To assess the changes in ever-screening and compliance at an individual level, we explored the flow across screening groups (ever-screened versus never, and compliant versus not) for all waves, using alluvial plots, which group categorical data into flows that can be traced across time points. Alluvial plots were constructed with memory, which tracked groups of individuals who had the same trajectories across the three waves. Plots were created for the overall population as well as for only those who had data at all three time points and were additionally stratified by location.

Finally, we investigated whether self-collection versus not, receipt of test result versus no receipt, and testing positive versus negative were associated with gaining or losing screening compliance. To investigate gaining compliance, the data was subset to baseline incompliant women. Those who screened during either follow-up wave were considered to have become compliant. Analogously, for loss of compliance, we subset to baseline compliant women. Those who had their last screen more than a year prior to enrollment and did not screen during the follow-up period were considered to have lost compliance. We ran multivariate logistic models to calculate odds ratios for each scenario. All models were adjusted for age (continuous), lifetime screening before study enrollment (yes/no), urbanicity (yes/no), ethnicity (Ladino, Tz’utujil, Q’eqchi’, Garífuna, other), and literacy (yes/no), in parallel with prior HPV MES analyses [19, 20].

## Results

**Fig. 1:**
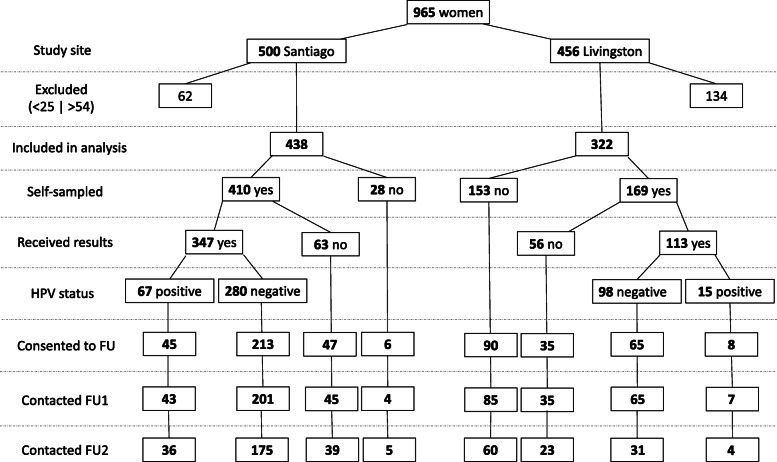
Flow chart of HPV MES participants included in analyses. “Study site” through “HPV status” include counts for the entire study population at baseline recruitment (excluding age ineligible women – see “Excluded <25 | > 54”), and “Consented to FU” through “Contacted FU2” include counts over the follow-up waves

The baseline wave of HPV MES included 956 women, of which 760 were age-eligible for this study. Of those, 509 consented to follow-up, and 485 (95%) and 373 (73%) were re-contacted in FU1 and FU2, respectively (354 (70%) contacted at both FU1 and FU2). Details of the study population can be found in Fig. 1. The population characteristics of the communities sampled for HPV MES were very similar to those in the nationally representative, Guatemalan Demographic and Health Surveys (DHS) data, published in 2017 [6], particularly when comparing to indigenous communities across the country (data not shown).

### Changes in ever-screened and screening compliance over follow-up

Table 1 presents the results of changes in reported ever-screening over follow-up. For most sub-groups, there is an increase in the percent of women who reported being ever-screened from baseline to FU1, but then a decrease from FU1 to FU2, which is logically impossible. This could have been due to the fact that the populations differed across waves (due to differential loss to follow-up across ever-screened groups). Thus, as a sensitivity analysis, we repeated these analyses with the subset of participants for who we had data at all three time points (Supplementary Material Table A1), finding similar increasing and then decreasing patterns across sub-groups.

**Table 1:** Changes in screening history among study participants over follow-up

	Ever screened			Compliant (screened past 3 years)		
	No. (%) – ever screened			No. (%) – screen compliant		
	Baseline	FU1	FU2	Baseline	FU1	FU2
	*n = 760*	*n = 477*	*n = 367*	*n = 760*	*n = 451*	*n = 367*
Overall	541 (71.2)	380 (79.7)	271 (73.8)	381 (50.1)	302 (67.0)	269 (73.3)
Age	*n = 760*	*n = 477*	*n = 367*	*n = 760*	*n = 451*	*n = 367*
25-39	339 (66.2)	252 (78.5)	180 (73.2)	242 (47.3)	208 (68.2)	179 (72.8)
40-54	202 (81.5)	128 (82.1)	91 (75.2)	139 (56.0)	94 (64.4)	90 (74.4)
Location	*n = 760*	*n = 477*	*n = 367*	*n = 760*	*n = 451*	*n = 367*
Santiago	316 (72.1)	241 (82.3)	195 (76.8)	206 (47.0)	183 (68.0)	207 (81.5)
Livingston	225 (70.0)	139 (75.5)	76 (67.3)	175 (54.3)	119 (65.4)	62 (54.9)
Ethnicty	*n = 747*	*n = 464*	*n = 354*	*n = 747*	*n = 444*	*n = 361*
Tz'utujil	299 (71.2)	234 (82.7)	187 (76.0)	194 (46.2)	178 (68.2)	201 (81.7)
Ladino	71 (75.5)	56 (87.5)	31 (70.5)	54 (57.4)	46 (73.0)	25 (56.8)
Q'echchi	71 (55.0)	55 (63.2)	19 (51.4)	49 (38.0)	48 (55.8)	14 (37.8)
Garifuna	88 (84.6)	30 (83.3)	28 (82.4)	76 (73.1)	27 (79.4)	24 (70.6)
Literacy	*n = 759*	*n = 476*	*n = 366*	*n = 759*	*n = 450*	*n = 366*
Reads and writes	352 (73.0)	243 (81.0)	166 (75.8)	256 (53.1)	194 (67.8)	150 (68.5)
Does not read or write	188 (67.9)	136 (77.3)	104 (70.7)	125 (45.1)	107 (65.2)	119 (81.0)
Education level	*n = 752*	*n = 473*	*n = 362*	*n = 752*	*n = 447*	*n = 362*
Less than primary	306 (68.6)	229 (77.9)	160 (71.1)	198 (44.5)	180 (65.2)	176 (78.2)
Primary or secondary	132 (75.4)	86 (80.0)	63 (76.8)	101 (57.7)	68 (67.3)	55 (67.1)
More than secondary	99 (75.6)	61 (85.9)	45 (81.8)	80 (61.1)	50 (71.4)	37 (67.3)
Collection status	*n = 760*	*n = 477*	*n = 367*	*n = 760*	*n = 451*	*n = 367*
Collected	421 (72.7)	316 (80.8)	230 (75.4)	293 (50.6)	250 (68.3)	237 (77.7)
No collect	120 (66.3)	64 (74.4)	41 (66.1)	88 (48.6)	52 (61.2)	32 (51.6)
Receipt status	*n = 579*	*n = 391*	*n = 305*	*n = 579*	*n = 366*	*n = 305*
Received results	336 (73.0)	259 (82.2)	190 (77.6)	230 (50.0)	210 (70.9)	190 (77.6)
No results	85 (71.4)	57 (75.0)	40 (66.7)	63 (52.9)	40 (57.1)	47 (78.3)
Results status	*n = 460*	*n = 315*	*n = 245*	*n = 460*	*n = 296*	*n = 245*
Received pos	59 (72.0)	46 (92.0)	33 (82.5)	40 (48.8)	41 (83.7)	30 (75.0)
Received neg	277 (73.3)	213 (80.4)	157 (76.6)	190 (50.4)	169 (68.4)	160 (78.0)

Changes in screening compliance over follow-up are also shown in Table 1. We see an overall gain in screening compliance of 16.9% from baseline to FU1 and 23.2% from baseline to FU2 (Table 1). However, the majority of the increase in screening compliance over follow-up was among Tz’utujil women from Santiago, with little change among the other ethnic groups. Women who identified as illiterate had a larger increase in screening compliance over follow-up (35.9% increase from baseline to FU2) compared to those who identified as literate (15.4% increase from baseline to FU2). Additionally, women who collected a sample for HPV testing had a larger increase in screening compliance over follow-up (27.1% increase from baseline to FU2) compared to those who did not (3.0% increase from baseline to FU2).

### Changes in recent screening behavior over follow-up

**Fig. 2:**
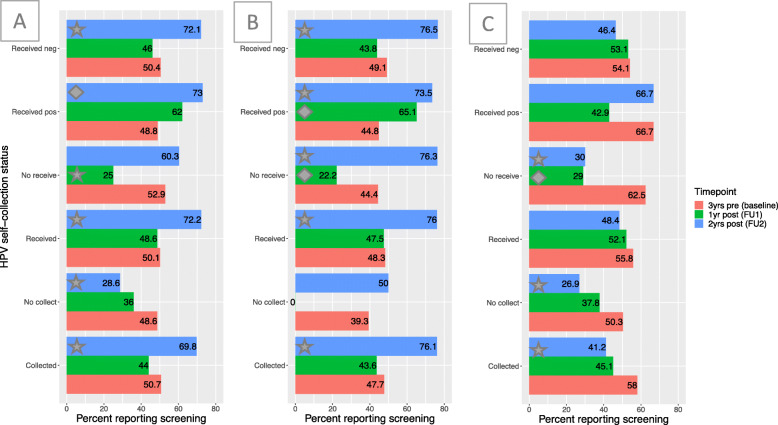
Changes in recent screening behavior over follow-up*.* The percent of women who report receiving cervical cancer screening over the following time intervals: within 3 years prior to study participation (at baseline), 1 year post-baseline participation in the study (at FU1), and 2 years post-baseline participation in the study (at FU2), stratified by the following (non-exclusive) groupings: those who chose to self-collect a sample to be tested for HPV (Collected), those who did not self-collect (No collect), those who received the results of their test (Received), those who did not receive results (No receive), those who received a positive HPV result (Received pos), and those who received a negative HPV result (Received neg). McNemar tests for pair proportions were used to compare baseline percentages to follow-up percentages. A star or diamond on a 1-year or 2-year follow-up bar represents a statistically significant change in percent from 3 years prior to baseline at the 0.05 and 0.1 level, respectively. **a** includes all eligible participants of HPV MES, **b** includes the subset from Santiago, and **c** includes the subset from Livingston

The changes in recent screening behavior from baseline to FU1 and FU2 among different populations can be seen in Fig. 2. For the overall population (Fig. 2a), there were statistically significant increases in the percent of women who reported screening between baseline and FU2 compared to the 3 years prior to baseline among those who self-collected (19.1% increase, *p* < 0.05), received results (22.1% increase, *p* < 0.05), and tested positive (24.2% increase, *p* < 0.1) or negative (21.7% increase, *p* < 0.05). In fact, there were no significant differences in the percent of women who reported screening in the 3 years before baseline compared to at FU1 (only 1 year after entry into the study), except among those who did not receive their results. The only groups that did not show a significant increase in screening at FU2 were those who did not receive their results and those who did not self-collect. We found similar results among the subset of participants who has data points at all three waves (*N* = 320), as shown in Supplementary Material Figure A1.

Comparable results were seen when restricting the analysis to the Santiago population (Fig. 2b). Those who self-collected (28.4% increase, *p* < 0.05), received results (27.7% increase, *p* < 0.05), and tested positive (28.7% increase, *p* < 0.05) or negative (27.4% increase, *p* < 0.05) had statistically significant increases in cervical cancer screening from 3 years prior to baseline to 2 years post (at FU2). Furthermore, among those who received a positive result, there was a significant increase in just the first year (FU1) after baseline (20.3% increase, *p* < 0.1) and those who did not receive their results had a significant increase by 2 years post baseline (31.9% increase, *p* < 0.05). Only those who did not self-collect, a small subset of this populations (*N* = 4 at FU1; *N* = 5 at FU2), did not have significant changes in screening behavior.

In contrast, in Livingston (Fig. 2c), where rates of reported screening prior to the study were higher, there were no significant increases over the follow-up period. We repeated this analysis by comparing literate to illiterate participants (Supplementary Figure A2), finding slightly larger screening increases over follow-up among women who identified as illiterate.

**Fig. 3:**
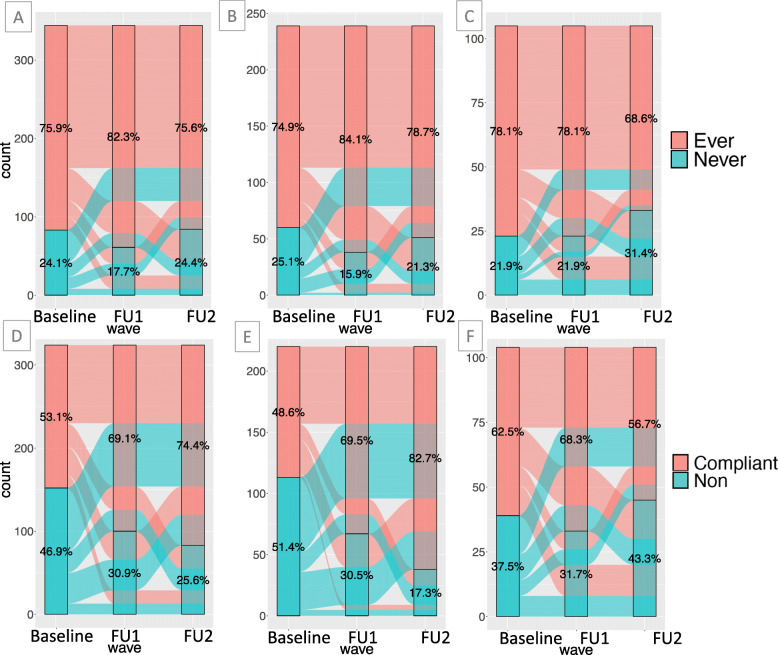
Alluvial plots of change in ever screened (**a-c**) and screen compliant (**d-e**). Ever screened is defined as at least one lifetime reported screen (ever/never), and screen compliant is reported screen in the past 3 years (compliant/non). For readability, only participants who had data at all three time points were included in these graphs (*N* = 344 for ever screened; *N* = 324 for screen compliant overall). **a** and **d** include all eligible participants, **b** and **e** include the subset from Santiago, and **c** and **f** include the subset from Livingston. Graphs include memory, thus movement between waves reflects individual screening histories

Figure 3 shows the transitions in individual screening histories (ever-screened and screen compliant for a three-year screening schedule) over follow-up waves. For clarity, figures include the subset of the study population who have data points for all three waves (*N* = 344 for ever-screened, *N* = 324 for screen complaint in the overall population). Exact counts for each exclusive screening trajectory group can be found in the Supplementary Material (Table A2), and figures including the entire study population can be found in the Supplementary Material (Figure A3). Figure 3a-c shows the movement of participants between ever- and never-screened in the overall population, Santiago subset, and Livingston subset, respectively; Fig. 3d-e show the movement of participants between screening compliant and non-compliant in the overall population, Santiago subset, and Livingston subset, respectively.

It does not appear that the overall number of women who reported ever-screening changed over the course of follow-up. Conversely, we do see a substantial number of women transitioning from non-compliant to compliant in the Santiago subpopulation at both FU1 and FU2, however, there does not appear to be a change among the Livingston subpopulation. As a sensitivity analysis, we compared women in Livingston who self-collected versus those who did not but found no difference (results not shown).

All transitions from ever- to never-screened reflect inconsistency in women’s reported screening histories. We defined an inconsistent screening history as reporting ever-screened at baseline and never-screened at FU1 or FU2, or ever-screened at FU1 and never-screened at FU2. Overall, 121 (16%) women (78 from Santiago and 43 from Livingston) had inconsistent screening histories and, of those who had ever-screened results at all three time points, 97 (28%) women (64 from Santiago and 33 from Livingston) had inconsistent screening histories.

The multivariate logistic regression analyses suggest that the odds of becoming screening compliant were higher for women who self-collected versus did not (AOR = 1.48, 95% CI = 0.64, 3.40), received their results versus did not (AOR = 1.29, 95% CI = 0.52, 3.02), or received a positive result versus negative (AOR = 2.43, 95% CI = 0.63, 16.10), although odds ratios were not statistically different from the null once the models were adjusted for relevant covariates (Table 2). As expected, the odds of losing Pap compliance were inversely associated with self-collection, receiving results, and tested positive, although again, these results were not statistically significant.

**Table 2:** Odds ratio of change in Pap compliancy given HPV testing choice and results

	Collected Sample ^a^		Received Results ^a^		Received Positive Result ^a^	
	OR	95% CI	OR	95% CI	OR	95% CI
Became Pap Compliant (Crude)^b^	3.01	(1.49, 5.98)	1.57	(0.67, 3.48)	2.71	(0.74, 17.57)
Became Pap Compliant (Adjusted)^c^	1.48	(0.64, 3.40)	1.29	(0.52, 3.02)	2.43	(0.63, 16.10)
Lost Pap Compliance (Crude)^b^	0.70	(0.37, 1.34)	0.86	(0.42, 1.80)	0.67	(0.24, 1.71)
Lost Pap Compliance (Adjusted)^c^	0.83	(0.40, 1.71)	0.94	(0.44, 2.04)	0.60	(0.21, 1.56)

^a^ Reference groups are: “No sample collected”, “Results not received”, and “Received negative result”, respectively

^b^ Became Pap compliance models, *N* = 256; Lost Pap compliance models, *N* = 238

^c^ Adjusted for age, Pap testing prior to enrollment in study, urbanicity, ethnicity, and literacy

## Discussion

The main objective of this study was to provide insight into the impact that the availability of HPV self-collection testing programs in rural and indigenous communities may have on overall ongoing engagement with existing cervical cancer screening programs.

Over the study follow-up period, overall reported cervical cancer screening rates increased, particularly among those who participated in the HPV self-collection campaign. In only 2 years after study entry, rates of screening among those who self-collected and received their results were statistically significantly higher than the 3 years prior to the study. However, most of this increase came from participants residing in Santiago; there was little change in behavior among participants from Livingston. Our prior work showed that women in Livingston had a much higher reported level of awareness of HPV at baseline and higher reported rates of screening prior to the study [19], which could partially explain the difference. We also found that women in the overall population were more likely to become screening compliant during follow-up if they had participated in HPV self-collection testing. Unfortunately, our analyses were complicated by (a) the fact that many women did not accurately report their screening history, as shown by women with inconsistent screening histories over the follow-up, and (b) that the women themselves chose to self-collect or not, rather than being randomized to receive the intervention.

This study has many strengths and provides new, unique information to the current literature on self-collection HPV testing programs. Prior studies from around the world [19, 20, 31–33] have found that self-collection HPV testing increases screening engagement in “hard-to-reach” or non-responding populations when used as an primary alternative to conventional screening [26, 34]. However, few have tracked women to see how self-collection HPV testing prospectively changes interaction with existing cervical cancer screening options. In communities where both screening and follow-up rates are low, using HPV self-testing to encourage participation in existing screening programs may be a useful intervention, but has not been explored prior to this study. Additionally, while our results are not generalizable to the overall Guatemalan population, our study population has similar characteristics to the overall rural and indigenous populations in Guatemala. Overall, we show the potential impact of an HPV testing program to improve rates of participation in national screening programs by increasing knowledge and empowerment among indigenous women, who tend to have low screening rates and high rates of cervical cancer.

These results should be understood within the context of the study’s limitations. As there are no available reporting systems for these types of data in our study setting, all data were self-reported, leading to inherent and well-documented problems with misreporting. This is clearly seen in the analyses, where ‘ever-screened’ was inconsistently reported by some participants. Probable causes for this reporting error include misremembering screening, misunderstanding screening (e.g. Pap or other screen versus a non-screening pelvic exam), and improved knowledge that causes a participant to realize they had previously misreported screening. The regularity of misreporting of cervical cancer screening history, particularly overreporting of ever-screening and frequency of screening, has been previously shown in the literature [35–37]. However, in circumstances where reporting systems are unavailable, it is important to collect data using feasible options. Screening compliance, which only necessitates accurate screening recall for the past few years, may thus be a more accurate measure of performance for screening programs than ever-screened.

Furthermore, research has shown that HPV negative women should not retest for a minimum of 3–5 years, due to the long-term safety of a negative HPV testing [17, 38]. It would thus be beneficial to investigate screening practices among HPV negative women 3–5 years post-baseline. Unfortunately, at this time there is only data for up to 2 years post-HPV testing.

This was an observational study where, due to ethical considerations, women were not randomized to receive HPV testing, resulting in non-comparable self-collection groups. We adjusted for potential confounders in the regression analyses, but there is still probable confounding. We also had small counts in some models, leading to wide confidence intervals and possible statistical imprecision, and we cannot make any strong causal inferences from these results. Finally, our self-collection versus no self-collection analyses are primarily comparisons among Livingston participants, as very few women chose not to self-collect in Santiago. We adjusted our models for location, but there is minimal information available from Santiago about non-self-collectors. This striking difference in results between the two communities demonstrates the need for tailored interventions, with a particular focus on how prior knowledge can inform a woman’s decision to participate in an HPV testing program.

## Conclusion

This study, one of the first of its kind, provides evidence to suggest that implementing a self-collection HPV testing program in underserved populations, particularly predominantly indigenous and rural communities, could improve engagement in existing cervical cancer screening programs. Our previous work suggests that the current availability of screening options (Pap smears or VIA) might affect how women perceive the need to try alternative programs, even if the available options are far from optimal [19]. While improving screening rates is critical to the prevention of cervical cancer in LMICs, high rates of screening will be meaningless without improvement of the quality of testing and follow-up and treatment rates. Future research needs to identify what community factors promote the success of HPV self-collection testing interventions, find new and efficient ways to educate women about cervical cancer prevention, link women to care after abnormal screening results, and to improve provider inclusion in these efforts.

## Supplementary information

**Additional file 1.** HPV MES baseline, FU1, FU2 data. Two tables and three figures providing results of sensitivity analyses conducted on main analyses; three blank surveys as used during data collection.

## Data Availability

The datasets used and/or analyzed during the current study are available from the corresponding author on reasonable request.

## Abbreviations

DHS: Demographic Health Survey
FU1: Follow-up wave 1 (2017)
FU2: Follow-up wave 2 (2018)
HIC: High-income country
HPV: Human papillomavirus
HPV MES: Human Papillomavirus Multi-Ethnic Study
LMIC: Low- and middle-income country
Pap: Papanicolaou test
VIA: Visual inspection with acetic acid
