# Supplementary material for: Using self-collection HPV testing to increase engagement in cervical cancer screening programs in rural Guatemala: a longitudinal analysis
Source: BMC Public Health. 2020 Sep 15;20:1406. doi: 10.1186/s12889-020-09478-8 (PMC7493167; doi:10.1186/s12889-020-09478-8)
Supplement: Supplementary file 1 — Additional file 1. HPV MES baseline, FU1, FU2 data. Two tables and three figures providing results of sensitivity analyses conducted on main analyses; three blank surveys as used during data collection. [file 12889_2020_9478_MOESM1_ESM.pdf]

Using Self-Collection HPV Testing to Increase Engagement in Cervical Cancer Screening Programs in Rural Guatemala:

Supplementary Material

Figure A1. Changes in screening behavior over follow-up among participants with complete data.
Figure A2. Changes in screening behavior over follow-up between literate and illiterate participants
Table A1. Changes in screening history among study participants with complete data over follow
Table A2. Percentage of participants by longitudinal screening status
Figure A3. Alluvial plots with overall population
Survey A1. Baseline survey
Survey A2. FU1 survey
Survey A3. FU2 survey

Figure A1. Changes in screening behavior over follow-up among participants with complete data. The percent of women who report receiving cervical cancer screening over the following time intervals: within three years prior to study participation (at baseline), one year post-baseline participation in the study (at FU1), and two years post-baseline participation in the study (at FU2), stratified by the following (non-exclusive) groupings: those who chose to self-collect a sample to be tested for HPV (Collected), those who did not self-collect (No collect), those who received the results of their test (Received), those who did not receive results (No receive), those who received a positive HPV result (Received pos), and those who received a negative HPV result (Received neg). The population is subset to only those individuals who had a reported screening history at all three timepoints (N = 320). McNemar tests for pair proportions were used to compare baseline percentages to follow-up percentages. A star and diamond on a 1-year or 2-year follow-up bar represent a statistically significant change in percent from three years prior to baseline at the 0.05 and 0.1 level, respectively. (A) includes all eligible participants of HPV MES, (B) includes the subset from Santiago, and (C) includes the subset from Livingston.

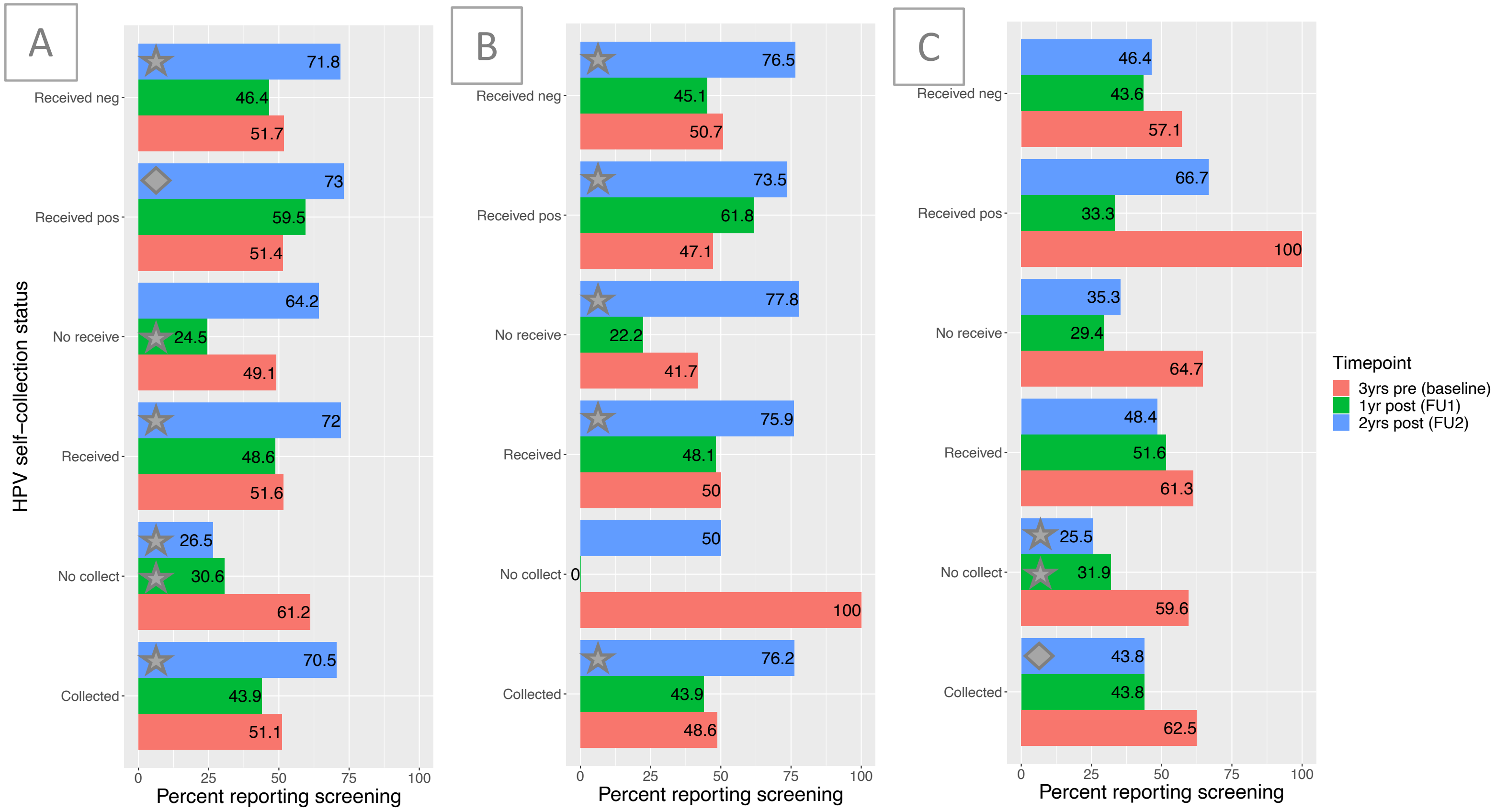

Figure A2. Changes in screening behavior over follow-up between literate and illiterate participants. The percent of women who report receiving cervical cancer screening over the following time intervals: within three years prior to study participation (at baseline), one year post-baseline participation in the study (at FU1), and two years post-baseline participation in the study (at FU2), stratified by the following (non-exclusive) groupings: those who chose to self-collect a sample to be tested for HPV (Collected), those who did not self-collect (No collect), those who received the results of their test (Received), those who did not receive results (No receive), those who received a positive HPV result (Received pos), and those who received a negative HPV result (Received neg). McNemar tests for pair proportions were used to compare baseline percentages to follow-up percentages. A star and diamond on a 1-year or 2-year follow-up bar represent a statistically significant change in percent from three years prior to baseline at the 0.05 and 0.1 level, respectively. (A) includes literate participants of HPV MES, (B) includes illiterate participants, and (C) includes literate participants who have data at all three time points and (D) includes illiterate participants who have data at all three time points.

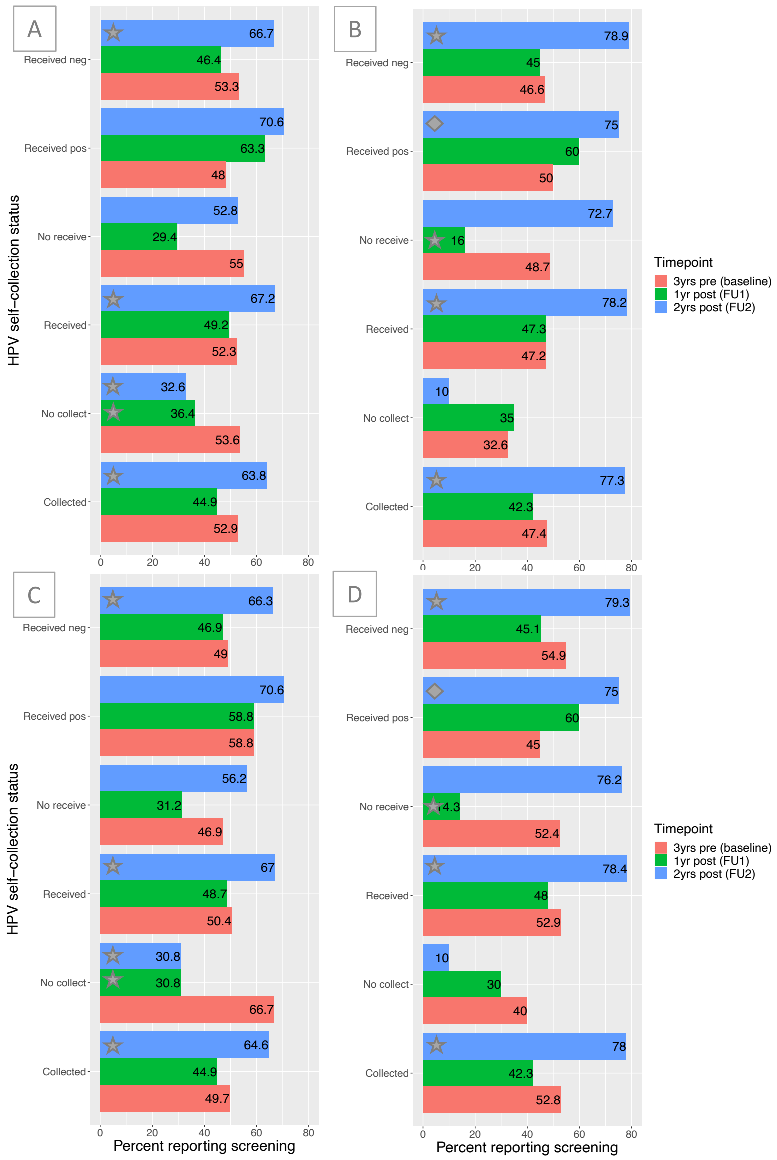

Table A1. Changes in screening history among study participants with complete data over follow

	Ever Screened (N = 344)			Compliant (screened past 3 yrs) (N = 324)		
	No. (%) – ever screened			No. (%) – screen compliant		
	Baseline	FU1	FU2	Baseline	FU1	FU2
	n = 344	*n = 344*	*n = 344*	*n = 324*	*n = 324*	*n = 324*
Overall	261 (75.9)	283 (82.3)	260 (75.6)	172 (53.1)	224 (69.1)	241 (74.4)
Age	*n = 344*	*n = 344*	*n = 344*	*n = 324*	*n = 324*	*n = 324*
25-39	159 (70.4)	184 (81.4)	170 (75.2)	106 (49.5)	153 (71.5)	158 (73.8)
40-54	102 (86.4)	99 (83.9)	90 (76.3)	66 (60.0)	71 (64.5)	83 (75.5)
Location	*n = 344*	*n = 344*	*n = 344*	*n = 324*	*n = 324*	*n = 324*
Santiago	179 (74.9)	201 (84.1)	188 (78.7)	107 (48.6)	153 (69.5)	182 (82.7)
Livingston	82 (78.1)	82 (78.1)	72 (68.6)	65 (62.5)	71 (68.3)	59 (56.7)
Ethnicty	*n = 339*	*n = 339*	*n = 339*	*n = 319*	*n = 319*	*n = 319*
Tz'utujil	171 (74.0)	195 (84.4)	180 (77.9)	104 (48.8)	148 (69.5)	177 (83.1)
Ladino	34 (79.1)	39 (90.7)	31 (72.1)	25 (58.1)	35 (81.4)	25 (58.1)
Q'echchi	24 (66.7)	22 (61.1)	19 (52.8)	20 (55.6)	18 (50.0)	14 (38.9)
Garifuna	28 (96.5)	23 (79.3)	25 (86.2)	22 (81.5)	20 (74.1)	21 (77.8)
Literacy	*n = 343*	*n = 343*	*n = 343*	*n = 323*	*n = 323*	*n = 323*
Reads and writes	161 (78.2)	174 (84.5)	159 (77.2)	107 (54.3)	139 (70.6)	138 (70.1)
Does not read or write	99 (72.3)	108 (78.8)	100 (73.0)	65 (51.6)	84 (66.7)	103 (81.7)
Education level	*n = 340*	*n = 340*	*n = 340*	*n = 320*	*n = 320*	*n = 320*
Less than primary	156 (73.2)	172 (80.8)	156 (73.2)	95 (48.0)	133 (67.2)	157 (79.3)
Primary or secondary	60 (81.1)	59 (79.7)	58 (78.4)	44 (62.9)	46 (65.7)	49 (70.0)
More than secondary	43 (81.1)	48 (90.6)	44 (83.0)	32 (61.5)	41 (78.8)	35 (67.3)
Collection status	*n = 344*	*n = 344*	*n = 344*	*n = 324*	*n = 324*	*n = 324*
Collected	219 (75.8)	242 (83.7)	223 (77.2)	140 (51.9)	190 (70.4)	213 (78.9)
No collect	42 (76.4)	41 (74.5)	37 (67.3)	32 (59.3)	34 (63.0)	28 (51.9)
Receipt status	*n = 289*	*n = 289*	*n = 289*	*n = 270*	*n = 270*	*n = 270*
Received results	181 (77.4)	202 (86.3)	184 (78.6)	116 (52.7)	163 (74.1)	173 (78.6)
No results	38 (69.1)	40 (72.7)	39 (70.9)	24 (48.0)	27 (54.0)	40 (80.0)
Results status	*n = 234*	*n = 234*	*n = 234*	*n = 220*	*n = 220*	*n = 220*
Received pos	27 (67.5)	38 (95.0)	33 (82.5)	21 (52.5)	34 (85.0)	30 (75.0)
Received neg	154 (79.4)	164 (84.5)	151 (77.8)	95 (52.8)	129 (71.7)	143 (79.4)

Table A2. Percentage of participants by longitudinal screening status

	Overall (N = 344)		Santiago (N = 239)		Livingston (N = 105)	
Ever versus Never Screened[A]	N	%	N	%	N	%
Ever-Ever-Ever	182	52.9	126	52.7	56	53.3
Ever-Ever-Never	41	11.9	30	12.6	11	10.5
Ever-Never-Ever	21	6.1	15	6.3	6	5.7
Never-Ever-Ever	42	12.2	34	14.2	8	7.6
Ever-Never-Never	17	4.9	8	3.3	9	8.6
Never-Ever-Never	18	5.2	11	4.6	7	6.7
Never-Never-Ever	15	4.4	13	5.4	2	1.9
Never-Never-Never	8	2.3	2	0.8	6	5.7
	Overall (N = 324)		Santiago (N = 220)		Livingston (N = 104)	
Compliant versus Non-Compliant Screening[A]	N	%	N	%	N	%
Comp-Comp-Comp	94	29.0	63	28.6	31	29.8
Comp-Comp-Non	28	8.6	13	5.9	15	14.4
Comp-Non-Comp	34	10.5	27	12.3	7	6.7
Non-Comp-Comp	76	23.5	61	27.7	15	14.4
Comp-Non-Non	16	4.9	4	1.8	12	11.5
Non-Comp-Non	26	8.0	16	7.3	10	9.6
Non-Non-Comp	37	11.4	31	14.1	6	5.8
Non-Non-Non	13	4.0	5	2.3	8	7.7

[A] Each row represents screening status at all waves of data collection (baseline, FU1, FU2).

Figure A3. Alluvial plots of change in *ever screened* (A-C) and *screen compliant* (D-E). *Ever screened* is defined as at least one lifetime reported screen (ever/never), and *screen compliant* is reported screen in the past three years (compliant/non). (A) and (D) include all eligible participants, (B) and (E) include the subset from Santiago, and (C) and (F) include the subset from Livingston. Graphs include memory, movement between waves reflects individual screening histories.

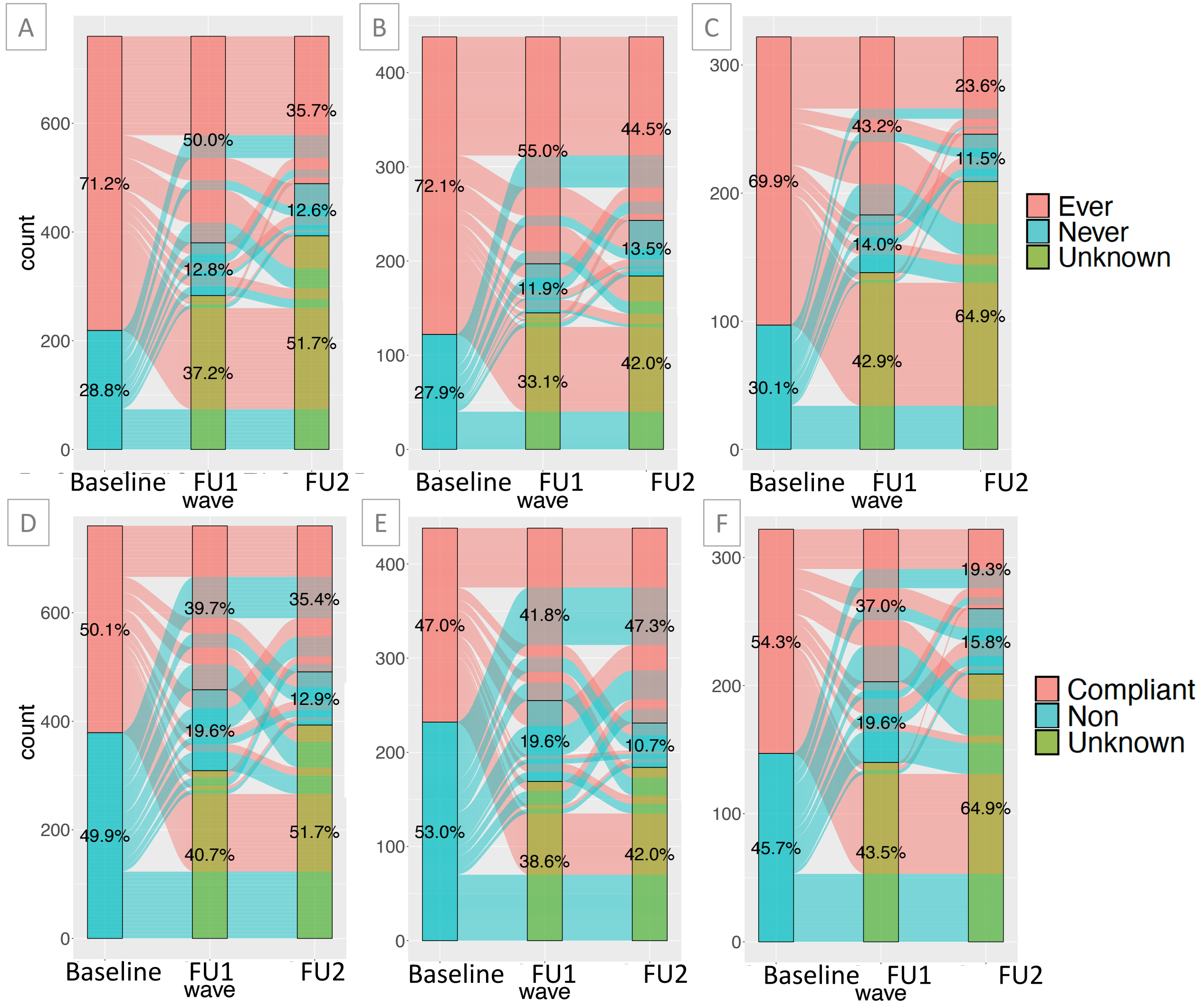

Baseline Survey

Question Text	Response Choices
How old are you?	Numeric
In what year were you born?	Numeric
With which one of the following ethnic groups do you identify yourself?	• Tz'tujil<br>• Ladino<br>• Garifuna<br>• Q'eqchi'<br>• Other (specify)<br>• Don't know/don't remember<br>• Decline to answer
Do you know how to read and write?	• Neither read nor write<br>• Read only<br>• Knows how to read and write<br>• Refuses to answer

What level of education have you reached?	• No formal schooling<br>• Primary school incomplete<br>• Primary school completed<br>• Secondary school completed<br>• Secondary school incomplete<br>• Technical school incomplete<br>• Technical school completed<br>• Pre-university incomplete<br>• Pre-University studies<br>• University incomplete<br>• University studies completed<br>• Post graduate work<br>• Don’t know/don’t understand<br>• Decline to answer
Have you ever been married or in a common law relationship?	• Yes<br>• No<br>• Refuses to respond
What is your marital status?	• Single<br>• Married<br>• Common law<br>• Separated<br>• Divorced<br>• Widowed<br>• Refuses to respond
How old were you when you got married or entered a common law relationship?	• Age (years)<br>• Don't know/ don't remember<br>• Decline to answer
Which of the following best describes your spouse or partner’s main job over the past few years?	• Government employee<br>• Private sector employee<br>• Independent worker<br>• Agricultural worker<br>• Unpaid worker<br>• Fishing<br>• Student<br>• Retired<br>• Unemployed (able to work)<br>• Unemployed (unable to work)<br>• Refuses to respond<br>• Other

Which of the following options best describes your main job over the past few years?	• Government employee<br>• Employed in private sector<br>• Independent worker<br>• Domestic Employee<br>• Textiles<br>• Artisans<br>• Agriculture<br>• Unpaid worker<br>• Fishing<br>• Student<br>• Retired<br>• Unemployed (able to work)<br>• Unemployed (unable to work)<br>• Refuses to Respond<br>• Other
Using the past year as a reference, what was the average income in your household? (Only answer one of the following)	• Per day<br>• Or per week<br>• Or per month<br>• Or per year<br>• Don’t know<br>• Decline to answer
Do you use any health services offered in or outside of your community?	• Yes<br>• No
What types of health services available in your community have you used? (Choose as many as you like)	• Puesto de Salud<br>• Centro de Salud<br>• Centro de Atención Médico Permanente (CAP)<br>• Centro de Atención Materno Infantil (CAIMI)<br>• Regional Hospital<br>• Private Clinic<br>• NGO Clinics<br>• Pharmacist<br>• Traditional Medicine<br>• Other (specify)<br>• Don't know/Don't remember<br>• Refuses to respond
What kind of services have you received from health care services? (Choose as many as apply)	• Informational Meeting<br>• Vaccination<br>• Birth control and family planning<br>• Delivery<br>• General Medicine<br>• Emergency<br>• Dentist<br>• Don't know/Don't remember<br>• Refused

What types of services provided in the previous health care services have you received? (Choose as many as apply)	• Educational meetings<br>• Vaccination<br>• Birth control and planning<br>• Delivery<br>• General Medicine<br>• Emergency<br>• Dentist
When was the last time you consulted a doctor?	• Less than a month ago<br>• Between 1 and 6 months<br>• Between 6 months and a year<br>• Between 1 and 2 years<br>• Between 2 and 3 years<br>• Between 3 and 4 years<br>• More than 5 years ago<br>• Never<br>• Don't know/ don't remember<br>• Decline to answer
Have you ever consulted with a traditional medicine practitioner when you were sick?	• Yes<br>• No<br>• Don't know/ don't remember<br>• Refused to respond
How many times have you visited a practitioner of traditional medicine because you were sick (approximately)?	• Only once<br>• 2-5 times<br>• 5-10 times<br>• Between 10 and 20 times<br>• More than 20 times
How many times do you consult with a traditional medicine practitioner for being sick?	• Always<br>• Almost always<br>• Sometimes<br>• Almost never<br>• Never
For what types of diseases do you consult with a traditional medicine practitioner?	Text entry
Where do you go when you want to consult with a doctor?	• A public clinic in your community<br>• A private clinic<br>• A traditional medicine practitioner<br>• A hospital in your community<br>• A hospital outside of your community<br>• Other (specify)<br>• Doesn't consult with a doctor<br>• Refuses to answer

<table>
<tr><td>When was the last time you consulted with a dentist?</td><td>

- Within the last 6 months
- Within the last year
- Within the last 5 years
- More than 5 years ago
- Never
- Don't know/ don't remember
- Refuses to answer

</td></tr>
<tr><td>Have you been informed by health care professional (doctor or nurse) that you have one of the following?</td><td>

	Yes	No	Don't know/Don't remember	Refuses to respond
Diabetes				
High Blood Pressure				
Heart Diseases				
High Cholesterol				
Other (specify)				

-

</td></tr>
<tr><td>The breast exam can be performed by you or a health professional. This exam consists of using the hands to check for the presence of masses or bumps on breasts. Have you ever heard of the breast auto-examination?</td><td>

- Yes
- No
- No sure
- Refuses to answer

</td></tr>
<tr><td>When was the last time a doctor performed a breast examination?</td><td>

- Less than a year ago
- 1-2 years
- More than 2 years
- Never
- Don't know/ Don't remember
- Refuses to answer

</td></tr>
<tr><td>Have you ever been taught how to do a self-breast examination?</td><td>

- Yes
- No
- Not sure
- Refuses to respond

</td></tr>
<tr><td>A mammogram is an exam to detect masses or abnormalities in women's breasts. Breasts are put in an x-ray machine in order to find these masses or abnormalities. Have you ever received a mammogram?</td><td>

- Yes
- No
- Don't know/ don't remember
- Refuses to respond

</td></tr>
<tr><td>When was the last time you received a mammogram?</td><td>

- Less than 1 year ago
- Less than 3 years ago
- Less than 5 years ago
- More than 5 years ago
- Don't know/ don't remember
- Refuses to respond

</td></tr>
</table>

<table>
<tr>
<td>Were you ever vaccinated against one of the following diseases? (If possible, use your vaccination card to answer this section).</td>
<td>
<table>
<tr><th></th><th>Yes</th><th>No</th><th>Don't know/ don't remember</th><th>Refuse</th></tr>
<tr><td>Hepatitis B</td><td></td><td></td><td></td><td></td></tr>
<tr><td>BCG/ Meningitis Tuberculosis</td><td></td><td></td><td></td><td></td></tr>
<tr><td>Rotavirus</td><td></td><td></td><td></td><td></td></tr>
<tr><td>OPV/ Poliomyelitis</td><td></td><td></td><td></td><td></td></tr>
<tr><td>Pentavalente (DPT/HB/Hib)</td><td></td><td></td><td></td><td></td></tr>
<tr><td>MMR</td><td></td><td></td><td></td><td></td></tr>
<tr><td>DPT</td><td></td><td></td><td></td><td></td></tr>
<tr><td>Influenza</td><td></td><td></td><td></td><td></td></tr>
<tr><td>Tetanus</td><td></td><td></td><td></td><td></td></tr>
</table>
</td>
</tr>
<tr>
<td>A Pap smear is a medical procedure that is used to observe the cells in the uterus and vagina. This procedure detects early abnormalities in the cervix, which can turn into cancer. The sample is obtained by a cervical scraping done by a doctor or nurse. Have you ever had a Pap smear?</td>
<td>• Yes
• No
• Don't know/ don't remember
• Refused to respond</td>
</tr>
<tr>
<td>When was the last time that you had a Pap smear?</td>
<td>• Within the last 6 months
• Within the last year
• Within the last 2 years
• Within the last 3 years
• Within the last 5 years
• More than 5 years ago
• Don't know/ don't remember
• Decline to answer</td>
</tr>
<tr>
<td>How many times have you had a Pap smear in your lifetime?</td>
<td>• 0
• 1-2
• 3-4
• 5 or more
• Don't know/ don't remember
• Refused to respond</td>
</tr>
<tr>
<td>Have you ever had an abnormal Pap smear result?</td>
<td>• Yes
• No
• The results were not reported back to me
• Don't know/ don't remember
• Decline to answer</td>
</tr>
</table>

How many times have you had an abnormal Pap smear result?	• 1 time<br>• 2 times<br>• 3 or more times<br>• Don't know/ don't remember<br>• Decline to answer
Did you receive any treatment for your abnormal Pap smear (cervical treatment)?	• Yes<br>• No<br>• Don't know/ don't remember<br>• Refused to respond
What is the main reason that you have never gotten a Pap smear?	• No reason/never thought about it<br>• Didn't need it/didn't know I needed this kind of test<br>• Haven't had any problems or reason<br>• Too expensive/no insurance/cost<br>• Too painful, unpleasant, embarrassing<br>• I don't have any knowledge of this test<br>• Other (specify)<br>• Don't know<br>• Refused to respond
Which of the following would encourage you to get screened for cervical cancer? (Choose as many as apply)	• Your doctor recommending it<br>• Being able to do the test yourself at home<br>• Being able to do it for free<br>• A friend or family member telling you why screening is important<br>• Other (specify)<br>• Refuses to respond
VIA, also known as the vinegar test, is a method to identify premalignant or malignant lesions in the cervix. During this test, acidic acid (vinegar) is applied to the uterus neck and if there are lesions, they will turn white. Have you ever had visual inspection using acetic acid (VIA)?	• Yes<br>• No<br>• Don't know/ don't remember<br>• Refuses to respond
When was the last time you were screened for cervical cancer using VIA?	• Less than a year ago<br>• 1 to 3 years ago<br>• 3 to 5 years ago<br>• More than 5 years ago<br>• Don't know/ don't remember<br>• Refuses to respond
How many times have you had a VIA?	• 0<br>• 1<br>• 2-3<br>• 4 or more
Has a doctor ever told you that you had genital warts?	• Yes<br>• No<br>• Don't know/Don't remember<br>• Refuses to respond

Has a doctor ever told you that you had premalignant lesions in your cervix?	• Yes<br>• No<br>• Don't know/Don't remember<br>• Refuses to respond
Has a doctor ever told you that you have cervical cancer?	• Yes<br>• No<br>• Don't know/ don't remember<br>• Refuses to respond
Have you ever been treated for malignant lesions or cervical cancer?	• Yes<br>• No<br>• Don't know/ don't remember<br>• Refuses to respond
Have you ever had any of the following tests?	(see sub-table below)
Have you been vaccinated for HPV (human papillomavirus)?	• Yes<br>• No<br>• Don't know/ don't remember<br>• Decline to answer
Have you ever smoked?	• Yes<br>• No<br>• Refuses<br>• Refuses to respond
How old were you when you started smoking?	Numeric
Do you currently smoke?	• Yes<br>• No<br>• Refused<br>• Refuses to respond
How many cigarettes do you smoke per day?	Numeric
How old were you when you quit smoking?	Numeric
How many cigarettes did you smoke per day during the time you smoked?	Numeric
Are you exposed to cigarette smoke?	• Yes<br>• No<br>• Don't know/Don't remember<br>• Refuses to respond
Do you use wood or coal in order to cook?	• Yes<br>• No<br>• Refuses to respond

	Yes	No	Don't know/Don't remember	Refuses to respond
Fecal blood test				
Colonoscopy				

Does your stove have a chimney or ventilation system?	• Yes<br>• No<br>• Don't know/Don't remember<br>• Refuses to respond
How much time a day do you spend cooking, approximately?	• Less than 30 minutes<br>• 30 minutes to an hour<br>• Between an hour and two hours<br>• Between two and three hours<br>• More than three hours<br>• Don’t know/Don’t remember<br>• Refuses to respond
Have you ever consumed alcohol?	• Yes<br>• No<br>• Refuses to respond
Do you drink alcohol regularly?	• Yes<br>• No<br>• Refuses to respond
During the past 30 days, on how many days did you have at least one drink of alcohol?	• 0<br>• 1-2<br>• 3-5<br>• 6-9<br>• 10-19<br>• 20-29<br>• Everyday<br>• Refuses to respond
During the past 30 days, on how many days did you have 5 or more drinks of alcohol in a row, meaning, within a couple of hours?	• 0<br>• 1<br>• 2<br>• 3-5<br>• 6-9<br>• 10-19<br>• 20 or more<br>• Decline to answer
Has anyone in your family ever been diagnosed with cervical cancer?	• Yes<br>• No<br>• Don't know/Don't remember<br>• Refuses to respond

Who in your family has been diagnosed with cervical cancer?	• Mother<br>• Grandmother<br>• Sister<br>• Daughter<br>• Cousin<br>• Niece<br>• Aunt<br>• Other (specify)<br>• Don't know/Don't remember<br>• Refuses to answer
Have you ever had sexual relations or any other type of sexual contact (with the mouth, hands, or contact with your vagina)?	• Yes<br>• No<br>• Decline to answer
Are you sexually active?	• Yes<br>• No<br>• Refuses to respond
Circumcision is defined as the surgical removal of the foreskin that covers the head of the penis. It is usually performed at the moment of birth or at a very young age. Is your current partner circumcised?	• Yes<br>• No<br>• Don't know/ don't remember<br>• Refuses to respond
How old were you when you first had vaginal intercourse?	• I've never had vaginal intercourse<br>• Age (numeric)<br>• Don't know/ don't remember<br>• Refuses to respond
How old was your partner when he had had vaginal intercourse for the first time?	• Age<br>• Don't know/ don't remember<br>• Decline to answer
Oral sex is defined as the stimulation of genitalia using the mouth (or any part of the oral cavity) or the throat (or any part of the oropharynx). Have you ever received or performed oral sex?	• Yes<br>• No<br>• Don't know/ don't remember<br>• Refuses to respond
With how many people have you had vaginal intercourse in the LAST SIX MONTHS?	Numeric
With how many people have you had vaginal intercourse in the LAST YEAR?	Numeric
With how many people have you had vaginal intercourse in your LIFETIME?	Numeric
How often do you use protection during vaginal intercourse?	• Always<br>• Almost always<br>• Sometimes<br>• Rarely<br>• Never<br>• Don't know/ don't remember<br>• Decline to answer

Please indicated if you have ever been tested for or diagnosed with any of the following sexually transmitted infections (STIs).	| | I have never been tested for it. | I have been tested for it, but never had it. | I have been diagnosed with it at least once. | Don't know. | Prefer not to answer. | HPV<br>Gonorrhea<br>Syphilis<br>Hepatitis B<br>Hepatitis C<br>HIV
Have you ever used oral contraceptives?	• Yes<br>• No<br>• Don't know/Don't remember<br>• Refuses to respond
For how many total years did you taken oral contraceptives?	• 0-6 months<br>• 6 months to a year<br>• 1-2 years<br>• more than 2 years<br>• Refuses to respond
Have you used an injected family planning method?	• Yes<br>• No<br>• Don't know/Don't remember<br>• Refuses to respond
How often to you get an injected family planning method?	• Every month<br>• Every 2 months<br>• Every 3 months<br>• Don't know/Don't remember<br>• Refuses to respond
How long have you been using an injected family planning method?	• 0-6 months<br>• 6 months to a year<br>• 1-2 years<br>• More than 2 years<br>• Don't know/Don't remember<br>• Refuses to respond

Have you ever used an IUD (Intrauterine Device)?	• Yes<br>• No<br>• Don't know/Don't remember<br>• Refuses to respond
For how many total years in total have you used an IUD?	• 0-6 months<br>• 6 months to a year<br>• 1-2 years<br>• More than 2 years<br>• Don't know/Don't remember<br>• Refuses to respond
Have you ever used any of these other types of contraceptives?	(see grid below)
How many times have you become pregnant (including abortions or miscarriages)?	• Numeric<br>• Refuses to respond
How many children do you have?	• Numeric<br>• Refuses to respond
How old were you when you had your first child?	Numeric
How serious is cervical cancer?	• Not at all<br>• A little<br>• Moderately<br>• Very serious<br>• Extremely serious

	Yes	No, but I have heard of it before	No, I have not heard of it before	Prefer not to answer	Don't know/Don't remember
Male Condom					
Female Condom					
Injection					
Patch					
Female Diaphragm					
Vaginal Ring					
Rhythm					
Breast Feeding					
Other (specify):					

Assuming you maintain the same lifestyle and health behaviors that you currently engage in, what are the chances that you will get cervical cancer in the future?	• Not likely<br>• Unlikely<br>• Moderately Probable<br>• Highly probable<br>• Surely<br>• Don't know
Who should be screened for cervical cancer? (Choose as many as apply)	• All women regardless of age<br>• All women beginning at puberty<br>• All women when they begin their sex lives<br>• Only women who are more sexually active<br>• Women with a family history of cervical cancer<br>• Only women with vaginal bleeding or discharge<br>• Older women<br>• Menopausal women<br>• Refuses to respond
If I were diagnosed with cervical cancer, it would strongly affect my life.	• Strongly Agree<br>• Agree<br>• Neutral<br>• Disagree<br>• Strongly Disagree<br>• Don't know/Unsure<br>• Refuses to respond
I believe that cervical cancer is a disease that lasts a long time.	• Strongly Agree<br>• Agree<br>• Neutral<br>• Disagree<br>• Strongly Disagree<br>• Don't know/Unsure<br>• Refuses to respond
If I were diagnosed with cervical cancer, I would have the ability to control it.	• Strongly Agree<br>• Agree<br>• Neutral<br>• Disagree<br>• Strongly Disagree<br>• Don't know/Unsure<br>• Refuses to respond
Cervical cancer is often curable with early detection and proper medical treatment.	• Strongly Agree<br>• Agree<br>• Neutral<br>• Disagree<br>• Strongly Disagree<br>• Don't know/Unsure<br>• Refuses to respond

If I developed cervical cancer, I would experience symptoms that would affect my life.	• Strongly Agree<br>• Agree<br>• Neutral<br>• Disagree<br>• Strongly Disagree<br>• Don't know/Unsure<br>• Refuses to respond
I believe that I am at risk for developing cervical cancer.	• Strongly Agree<br>• Agree<br>• Neutral<br>• Disagree<br>• Strongly Disagree<br>• Don't know/Unsure<br>• Refuses to respond
Being diagnosed with cervical cancer would affect me emotionally (for example, it would make you angry, scared, upset or depressed).	• Strongly Agree<br>• Agree<br>• Neutral<br>• Disagree<br>• Strongly Disagree<br>• Don't know/Unsure<br>• Refuses to respond
If I were diagnosed with cervical cancer, which would be the three most probable causes?	• The most probable (text entry)<br>• The second most probable (text entry)<br>• The third most probable (text entry)
Before today, had you heard of HPV (human papillomavirus)?	• Yes<br>• No<br>• Refuses to respond
Have you ever heard there is a test to find HPV?	• Yes<br>• No<br>• Don't know
How many times have you been tested for HPV?	• 0<br>• 1<br>• 2<br>• 3-5<br>• More than 5
What was your age at your last test for HPV?	Numeric
Has a doctor ever told you that you have HPV?	• Yes<br>• No<br>• Don't know/Don't remember<br>• Refuses to respond

Have you heard that HPV can lead to the following conditions? (choose all the apply)	• Genital warts<br>• Warts in the oral cavity or oropharynx<br>• RRP (recurrent respiratory papillomatosis)<br>• Cervical cancer<br>• Anal cancer<br>• Other genital cancers<br>• Oropharyngeal cancer<br>• Other head and neck cancers<br>• I have not heard that HPV can lead to any of these conditions
I feel I am at risk of having HPV.	• Yes<br>• No<br>• Don't know/Don't remember<br>• Refuses to respond
Do you think that HPV is a sexually transmitted disease?	• Yes<br>• No<br>• Don't know/Don't remember<br>• Refuses to respond
I feel I should get tested for HPV.	• Yes<br>• No<br>• Don't know/Don't remember<br>• Refuses to respond
If present, HPV… (Check ALL that apply)	• Can cause cervical cancer<br>• Can cause infertility<br>• Can cause warts<br>• Will usually disappear in its own<br>• Can cause death<br>• Can cause sterility<br>• Don't know
Which of the following increases your risk for HPV infection? (Circle ALL that apply)	• If you begin your sex life at an early age<br>• If you have many sexual partners<br>• If your partner has many sexual partners<br>• Birth control pills<br>• Smoking<br>• Excessive stress<br>• Poor nutrition<br>• Don't know
Have you heard that there is a vaccine against HPV?	• Yes<br>• No<br>• Refuses to respond
I believe that the HPV vaccine prevents most genital warts.	• Yes<br>• No<br>• Refuses to respond

I believe that the HPV vaccine prevents most cervical cancer.	• Yes<br>• No<br>• Refuses to respond
HPV is one of the main risk factors for cervical cancer. It is transmitted sexually and there exists a vaccine that prevents the most common strains of the virus.	• Yes<br>• No<br>• Don't know/Don't remember<br>• Refuses to respond
If an HPV vaccine were available in Guatemala, would you get it?	• Yes<br>• No<br>• Don't know<br>• Decline to answer
Would you vaccinate your daughters against HPV?	• Yes<br>• No<br>• I don't have any daughters<br>• Don't know/Don't remember<br>• Decline to answer
What concerns would you have about getting the HPV vaccine? Name up to three concerns.	• Concern #1 (text response)<br>• Concern #2 (text response)<br>• Concern #3 (text response)<br>• None
HPV is the main cause of the majority of cervical cancers. Therefore, the HPV test is being considered as a prevention and early detection strategy against cervical cancer. There are kits that allow you to take the sample yourself in the privacy of your own home to collect a sample and then mail in the sample to be tested for HPV.<br><br>Where would you prefer to have a test for HPV performed?	• At home<br>• At a doctor's office<br>• Don't know/ No response
Who would you prefer to perform the swab for the HPV test?	• My doctor<br>• Myself<br>• Don't know/ no response
I would be willing to collect a sample at home.	• Yes<br>• No<br>• Don't know/ no response
I would be embarrassed to collect a sample at home.	• Yes<br>• No<br>• Don't know/ no response
I am worried that I would not collect the sample properly.	• Yes<br>• No<br>• Don't know/ no response
I am afraid that HPV testing will show that I have cervical cancer.	• Yes<br>• No<br>• Don't know/ no response

I am afraid that HPV testing will make other people think that I have cervical cancer.	• Yes<br>• No<br>• Don't know/ no response
I am worried the self-sampling will be painful.	• Yes<br>• No<br>• Don't know/ no response
I would be willing to have a health worker drop off a swab to take a self-collection HPV sample.	• Yes<br>• No<br>• Don't know/ no response
I would need my husband/partner's approval to collect the sample.	• I don't have a husband/partner<br>• Yes<br>• No<br>• Don't know/ no response
I would be willing to go to the nearest health center to drop off the swab.	• Yes<br>• No<br>• Don't know/ no response
My religion/spiritual belief would affect my decision to be screened.	• Yes<br>• No<br>• Don't know/ no response
I believe it is necessary to be screened for cervical cancer.	• Yes<br>• No<br>• Don't know/ no response
I would be willing to try an HPV self-collection test (using a kit provided by the study).	• Yes<br>• No<br>• Don't know/ no response
I would be willing to go to the clinic for a pelvic examination and Pap smear if the self-collected sample was found abnormal.	• Yes<br>• No<br>• Don't know/ no response

Question Text	Response Choices

How old were you when you had your first period?	Numeric
Did you ever receive a Pap/VIA before participation in this study?	• Yes<br>• No<br>• Don't know/don't remember
Why have you never received a Pap/VIA prior to participation in this study?	• Didn't know about it<br>• Didn't know I needed one<br>• Too expensive without voucher<br>• I was unable to attend a clinic<br>• My family doesn't allow me to get one<br>• I would be ashamed<br>• Other
When was the last time you received a Pap/VIA before participation in this study?	• Less than 1 year ago<br>• 1-3 years ago<br>• More than 3 years ago<br>• Don't know/don't remember
Approximately how many Pap/VIAs have you received in your lifetime?	• 1-2<br>• 3-4<br>• 5-6<br>• 7 or more<br>• Don't know/don't remember
Did you choose to perform a self-collection HPV test during your participation in the study?	• Yes<br>• No
Did you receive the results of this test?	• Yes<br>• No
What were the results of your HPV test?	• Positive<br>• Negative<br>• Don't know/don't remember
How would you rate the quality of the explanation you received of your HPV results?	• Very satisfactory<br>• Satisfactory<br>• Neutral<br>• Poor<br>• Very poor
Did you follow the recommendations provided to you with your results?	• Yes<br>• No<br>• Unsure/don't know
Have you received a Pap/VIA since your participation in this study?	• Yes<br>• No
Why did you choose to receive a Pap/VIA since you started participating in the study, when you have never received one before?	• Didn't know about it before study<br>• Didn't know I needed one before study<br>• Too expensive without voucher<br>• Other

Where did you receive the Pap/VIA?	• CAIMI<br>• Fundaeco<br>• Rxiin Tnamet<br>• Private doctor/hospital<br>• Jornada de Salud<br>• Other
How would you rate your experience at the location where you received your Pap/VIA?	• Very satisfactory<br>• Satisfactory<br>• Neutral<br>• Poor<br>• Very poor
Did you use your voucher provided by the study?	• Yes<br>• No
Were you informed of the results of your Pap/VIA?	• Yes<br>• No
How were you informed of your results?	• Phone call<br>• In person
What were the results of your Pap/VIA?	• Normal/negative/all fine<br>• Abnormal/positive/need follow-up<br>• Don't know
Were you recommended for further follow-up?	• Yes<br>• No
What follow-up was recommended?	• Colposcopy<br>• Biopsy<br>• Cryotherapy<br>• Follow-up testing<br>• Cancer referral<br>• Don't know/don't remember
What institution were you referred to for this follow-up?	• CAIMI<br>• Hospital Nacional (de Solola o de la Amistad Japon Guatemala)<br>• APROFAM<br>• INCAN<br>• Private doctor/hospital<br>• Other
Have you scheduled the recommended follow-up?	• Yes<br>• No
Have you attended the visit and received the results from the follow-up?	• Yes<br>• No
What were the results of the recommended follow-up?	• Normal/negative/all fine<br>• Abnormal/positive/needs another follow up<br>• Don't know

What is the most important reason for why you have not scheduled the recommended follow-up?	• Haven't had time<br>• Don't know where to go<br>• Don't have the money<br>• Don't think it is important<br>• My family didn't want me to go<br>• The place that I need to go is too far away<br>• Other
When are you planning to get your next cervical cancer screening test?	• The next time that I go to the doctor<br>• Within a year<br>• Within the next two years<br>• More than 2 years from now<br>• Unsure/Don't know<br>• When there is another opportunity
Why did you choose not get a Pap/VIA?	• I haven't had time<br>• I tested positive for HPV, but don't think I need one<br>• I tested negative for HPV, so don't think I need one<br>• My family doesn't want me to get one<br>• Other
Are you planning to get a Pap/VIA in the next 6 months?	• Yes<br>• No
Why are you not planning to get a Pap/VIA?	• I won't have time<br>• I don't need one<br>• My family doesn't want me to get one<br>• Other

Question Text	Response Choices

Have you ever had a Pap/VIA?	• No<br>• Yes
At what age did you have your first Pap/VIA?	Numeric
How often do you get a Pap/VIA exam?	• Every year or more frequently<br>• Every 1-3 years<br>• More than 3 years<br>• I have had it only once or twice
Approximately how many Pap/VIAs have you received in your lifetime?	Numeric
At what age did you have your last Pap/VIA?	Numeric
Where did you get your last Pap/VIA?	• Hospital<br>• Centro de salud<br>• Puesto de salud<br>• Otro público<br>• Clínica / hospital privado<br>• Consultorio médico<br>• APROFAM<br>• Farmacia<br>• IGSS<br>• Otro privado<br>• Sin información
Did you get it at any of the following?	• CAIMI<br>• Hospital Nacional (de Solola o de la Amistad Japon Guatemala)<br>• APROFAM<br>• INCAN<br>• Fundaeco<br>• Rxiin Tnamet<br>• Private doctor/hospital<br>• Jornada de Salud<br>• I did not get it at any of the above

Which of the following reasons contributed to you never receiving a Pap/VIA?	• Did not know about it<br>• Did not know I needed one<br>• Too expensive<br>• Clinic is too far<br>• My family does not allow me to get one<br>• I would be ashamed<br>• I would not want to go alone<br>• I am afraid to have a Pap/VIA because I might find out something is wrong<br>• I am afraid to have a Pap/VIA because I do not understand what will be done<br>• I do not know how to go about getting a Pap/VIA<br>• Concerned that there may not be a provider at the clinic<br>• Concerned that there may not be a female health provider at the clinic<br>• Having a Pap/VIA is too embarrassing<br>• Having a Pap/VIA takes too much time<br>• Concerned that there may not be Pap/VIA available at the clinic<br>• Having a Pap/VIA is too painful<br>• People doing Pap/VIA are rude to women<br>• Having a Pap/VIA exposes me to unnecessary radiation<br>• I cannot remember to schedule a Pap/VIA<br>• I have other things to do more important than getting a Pap/VIA<br>• I am too old<br>• I am afraid that getting a Pap/VIA would get me pregnant<br>• None of these apply
Did you have Pap/VIA within the past two years (since June 2016 when you first participated in our study)?	• No<br>• Yes<br>• Don't know<br>• Don't remember<br>• Refused to answer
Did you have Pap/VIA within the past year (since June 2017 or the last time we talked)?	• No<br>• Yes<br>• Don't know<br>• Don't remember<br>• Refused to answer

Why did you choose to get your last Pap/VIA?	• My doctor recommended it<br>• It was my time (Me tocaba)<br>• My friends/relatives recommended it<br>• I did not have to go by myself<br>• I learned from the study that I should do it<br>• Because of the test I got as part of the study<br>• Because I was positive for the HPV test<br>• Because the study provided me with a voucher<br>• I knew it was offered at the clinic<br>• There was a female health provider at the clinic<br>• I knew there would be a Pap/VIA available at the clinic<br>• As a follow-up of another Pap/VIA exam<br>• None of these apply to me
Were you informed of the results of your last Pap/VIA?	• No<br>• Yes<br>• Don't know<br>• Don't remember<br>• Refused to answer
How were you informed of your results of your last Pap/VIA?	• Phone call<br>• In person<br>• Community health care worker<br>• Don't remember
What were the results of your Pap/VIA?	• Normal/negative/all fine<br>• Abnormal/positive/need follow-up<br>• Don't know<br>• Don't remember<br>• Refused to answer
Were you recommended for further follow-up?	• No<br>• Yes<br>• Don't know<br>• Don't remember<br>• Refused to answer
What follow-up was recommended?	• Colposcopy<br>• Biopsy<br>• Cryotherapy<br>• Follow-up testing<br>• Cancer referral<br>• Another Pap/VIA<br>• Don't know<br>• Don't remember<br>• Refused to answer

What institution were you referred to for this follow-up?	• CAIMI<br>• Hospital Nacional (de Solola o de la Amistad Japon Guatemala)<br>• APROFAM<br>• INCAN<br>• Private doctor/hospital<br>• Fundaeco<br>• Rxiin Tnamet<br>• Centro de salud<br>• Puesto de salud<br>• Jornada de Salud<br>• Clínica / hospital privado<br>• Consultorio médico<br>• Farmacia<br>• IGSS<br>• None of these institutions<br>• Don't know<br>• Don't remember<br>• Refused to answer
Have you scheduled the recommended follow-up?	• No<br>• Yes<br>• Don't know<br>• Don't remember<br>• Refused to answer
Have you attended the visit and received the results from the follow-up?	• No<br>• Yes<br>• Don't know<br>• Don't remember<br>• Refused to answer
What were the results of the recommended follow-up?	• Normal/negative/all fine<br>• Abnormal/positive/needs another follow up<br>• Don't know<br>• Don't remember<br>• Refused to answer

What is the most important reason for why you have not scheduled the recommended follow-up?	• Did not know about it<br>• Do not know where to go<br>• Did not know I needed one<br>• Do not think it is important<br>• Have not had time<br>• Too expensive<br>• Clinic is too far<br>• My family does not allow me to get one<br>• I would be ashamed<br>• I would not want to go alone<br>• I am afraid to have a Pap/VIA because I might find out something is wrong<br>• I am afraid to have a Pap/VIA because I do not understand what will be done<br>• I do not know how to go about getting a Pap/VIA<br>• Concerned that there may not be a provider at the clinic<br>• Concerned that there may not be a female health provider at the clinic<br>• Having a Pap/VIA is too embarrassing<br>• Having a Pap/VIA takes too much time<br>• Concerned that there may not be Pap/VIA available at the clinic<br>• Having a Pap/VIA is too painful<br>• People doing Pap/VIA are rude to women<br>• Having a Pap/VIA exposes me to unnecessary radiation<br>• I cannot remember to schedule a Pap/VIA<br>• I have other problems more important than getting a Pap/VIA<br>• I am too old to need a routine Pap/VIA<br>• I am afraid that getting a Pap/VIA would get me pregnant<br>• I am pregnant<br>• None of these apply
When are you planning to get your next cervical cancer screening test?	• The next time that I go to the doctor<br>• Within a year<br>• Within the next two years<br>• More than 2 years from now<br>• When there is another jornada<br>• Unsure/Don't know

Why did you choose not get a Pap/VIA within the past two years?	• I have not had time<br>• I tested positive for HPV, but do not think I need one<br>• I tested negative for HPV, so do not think I need one<br>• My family does not want me to get one<br>• I am afraid to have a Pap/VIA because I might find out something is wrong<br>• I am afraid to have a Pap/VIA because I do not understand what will be done<br>• I do not know how to go about getting a Pap/VIA<br>• Having a Pap/VIA is too embarrassing<br>• Having a Pap/VIA takes too much time<br>• Having a Pap/VIA is too painful<br>• People doing Pap/VIA are rude to women<br>• Having a Pap/VIA exposes me to unnecessary radiation<br>• I cannot remember to schedule a Pap/VIA<br>• I have other problems more important than getting a Pap/VIA<br>• I am too old to need a routine Pap/VIA<br>• I am afraid that getting a Pap/VIA would get me pregnant<br>• I was pregnant<br>• None of these apply to me
Are you planning to get a Pap/VIA in the next year?	• No<br>• Yes<br>• Don't Know
Why are you not planning to get a Pap/VIA?	• I don't have time<br>• I do not need one<br>• My family does not want me to get one<br>• I am afraid to have a Pap/VIA because I might find out something is wrong<br>• I am afraid to have a Pap/VIA because I do not understand what will be done<br>• I do not know how to go about getting a Pap/VIA<br>• Having a Pap/VIA is too embarrassing<br>• Having a Pap/VIA takes too much time<br>• Having a Pap/VIA is too painful<br>• People doing Pap/VIA are rude to women<br>• Having a Pap/VIA exposes me to unnecessary radiation<br>• I cannot remember to schedule a Pap/VIA<br>• I have other problems more important than getting a Pap/VIA<br>• I am too old to need a routine Pap/VIA<br>• I am afraid that getting a Pap/VIA would get me pregnant<br>• I am pregnant<br>• None of these apply to me
